# Indonesia’s forest conversion moratorium assessed with an agent-based model of Land-Use Change and Ecosystem Services (LUCES)

**DOI:** 10.1007/s11027-016-9721-0

**Published:** 2016-08-14

**Authors:** Aritta Suwarno, Meine van Noordwijk, Hans-Peter Weikard, Desi Suyamto

**Affiliations:** 1 Environmental Systems Analysis Group, Wageningen University, Lumen Building, building no. 100, room A. 235, Droevendaalsesteeg 3, 6708PB, Wageningen, the Netherlands; 2 World Agroforestry Centre (ICRAF), SE Asia program, Bogor, Indonesia; 3 Plant Production Systems Group, Wageningen University, Wageningen, the Netherlands; 4 Environmental Economics and Natural Resources Group, Wageningen University, Wageningen, the Netherlands; 5 Center for International Forestry Research (CIFOR), Bogor, Indonesia

**Keywords:** Agent-based model, Central Kalimantan, Climate mitigation, Decision-making, Carbon emissions, Households, Land-use change, Private companies

## Abstract

The Indonesian government recently confirmed its Intended Nationally Determined Contributions (INDCs) to mitigate global climate change. A forest moratorium policy that protects forest and peatland is a significant part of the INDCs; however, its effectiveness is unclear in the face of complex land-use and land-cover change. This study aims to assess the dynamics of land-use change and ecosystem service supply as a function of local decision-making. We developed an agent-based model, Land-Use Change and Ecosystem Services (LUCES), and used it to explore the possible effects of the forest moratorium policy on the land-use decisions of private companies and communities. Our simulations for two districts in Central Kalimantan show that the current implementation of the forest moratorium policy is not effective in reducing forest conversion and carbon emissions. This is because companies continue to invest in converting secondary forest on mineral soils and the moratorium does not affect community decision-making. A policy that combines a forest moratorium with livelihood support and increases farm-gate prices of forest and agroforestry products could increase the local communities’ benefits from conservation. Forest and agroforestry areas that are profitable and competitive are more likely to be conserved and reduce potential carbon emission by about 36 %. The results for the two districts, with different pressures on local resources, suggest that appropriate additional measures require local fine-tuning. The LUCES model could be an ex ante tool to facilitate such fine-tuning and help the Indonesian government achieve its INDC goals as part of a wider sustainable development policy.

## Introduction

A landmark agreement in combating climate change was achieved at the Conference of the Parties (COP) 21 of the UN Framework Convention on Climate Change (UNFCCC) in Paris (UNFCCC 2015a, b). This agreement charted a new course in the global effort to enhance support and assistance for developing countries to combat climate change and to adapt to its effects. The Paris Agreement’s central aim is to strengthen the global response to the climate change threats and the ability of countries to deal with the impacts of climate change. In the preparation of the agreement, the countries involved agreed to publicly outline what post-2020 climate actions they intend to take under a new international agreement, known as their Intended Nationally Determined Contributions (INDCs). The INDCs will largely determine whether a path towards a low-carbon, climate-resilient future is feasible. INDCs link national climate policy targets to a global framework that drives collective climate action. INDCs should also articulate how a country is integrating climate change into other national priorities, such as sustainable development and poverty reduction, and encourages the private sector to contribute to these efforts (UNFCCC 2015a).

Indonesia, as one of the countries that has already submitted its INDCs, has outlined its transition to a low-carbon emission future, describing the enhanced actions and necessary efforts to prevent a 2 °C increase in global temperature (UNFCCC 2015b). Initiatives to reduce carbon (CO_2_) emissions started in 2009 when Indonesia voluntarily pledged to unconditionally reduce 26 % of its projected greenhouse gases under a business-as-usual scenario by 2020. Conditional on international support, a 41 % emission reduction was deemed possible (Howson and Kindon 2015; Yamamoto and Takeuchi 2016). In the INDCs, the estimates were revised to meet a 29 % reduction by 2030 compared with the business as usual scenario where a 41 % reduction is feasible with international support. Since 2009, Indonesia has progressed steadily to formulate legal and policy instruments to support this commitment. One significant step was a moratorium on primary forest clearance and peatland conversion from 2010 to 2016 to reduce emissions from Land-Use, Land-Use Change and Forestry (LULUCF) and to restore the benefits from forest ecosystems (McNeish et al. 2011; Astuti and McGregor 2015). This policy is also aimed at improving transparency in forest governance that could be seen as the means to establish enabling conditions to reduce the emissions from LULUCF (Murdiyarso et al. 2011). It clearly states that new concessions for primary and peat forest conversion will not be issued. Moreover, an integrated forestry map would be produced. Actions and investments in a sustainable low-carbon emission future under the forest moratorium are important to protect high terrestrial carbon stocks. However, the moratorium as such does not address livelihood options for forest dependent people. This exclusion has caused difficulties in implementing the policy (Sloan 2014) together with unresolved contests over land tenure (Galudra et al. 2011; Sloan et al. 2012; van Noordwijk et al. 2014).

Several studies have been conducted to explore the effectiveness of the forest moratorium in decelerating land-use change and forest conversion (Sloan et al. 2012; Sloan 2014; Margono et al. 2014; Astuti and McGregor 2015; Busch et al. 2015). In these studies, the effectiveness of the forest moratorium is analysed by comparing the rate of land-use change and forest conversion before and after the implementation of the policy. They highlight options to improve the capacity of local and national governments (Sloan et al. 2012; Sloan 2014) by monitoring systems (Margono et al. 2014; Astuti and McGregor 2015) or carbon pricing (Busch et al. 2015) to make a forest moratorium work towards decelerating land-use change. However, the option of improving the effectiveness of a forest moratorium through sustainable ecosystem benefits and support for local livelihoods has not been considered.

The aim of this study was to model land-use change and ecosystem service supply, including CO_2_ storage, in two Indonesian districts and to explore how forest moratorium policies influence change in the land-use decisions of companies and communities. As a tool for this analysis, we developed an agent-based Land-Use Change and Ecosystem Services (LUCES) model to capture the human-environment system in tropical forest margins. The LUCES model is a hybrid model that provides a comprehensive representation of the coupled socio-ecological system. It was developed and calibrated for two districts in Central Kalimantan Province to address the integration of local community (household) and private company decision-making in response to the forest moratorium policies and the impact of these decisions on the capacity of ecosystems to provide provisioning and regulating services. The two districts were selected based on the differences in local community composition, migration history, population density and history of natural resource extraction (Suwarno et al. 2015). These differences were assumed to have influenced the decisions of communities and private companies to change land-use. This will ultimately have an impact on forest ecosystems and CO_2_ emissions. In the context of Indonesia’s INDCs, the results of this study will support the design of additional programmes for effective forest moratorium policies that reduce emissions from LULUCF and sustain local livelihoods.

## Methods

### Site description

**Fig. 1:**
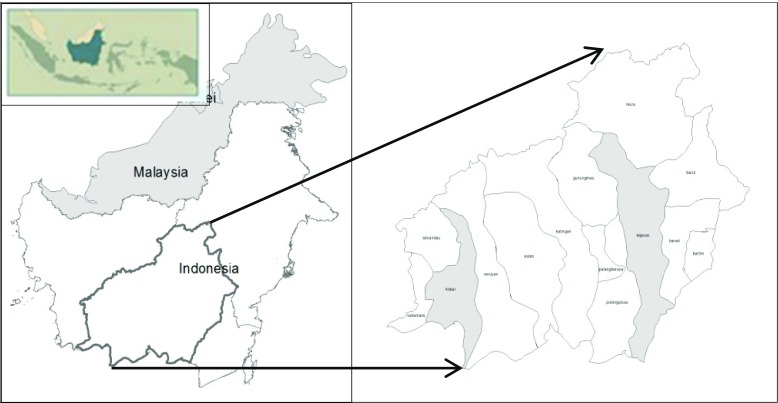
Case study area in the districts of West Kotawaringin and Kapuas (*highlighted in grey*)

This study was conducted for West Kotawaringin and Kapuas districts in Central Kalimantan Province (Fig. 1). These two districts have experienced different natural resource management, which still influence the perceptions and expectations of local people and the district governments.

West Kotawaringin district is situated in the western part of Central Kalimantan with a total area of about 8381 km^2^. The district has a population density of about 28 people/km^2^ with an annual population growth rate of 4.2 % (see Table 1). Timber (from natural forest and forest plantations) has been the main livelihood of the local people for almost two decades, starting around the 1980s. The boom in the timber industry provided sufficient income not only for local people but also for the district government. In the two following decades, West Kotawaringin became one of the richest districts in Central Kalimantan (National Statistic Bureau (BPS) 2013). The collapse of the logging/timber industry in the mid 2000s and the increase in international palm oil prices have driven logging companies to shift their business to oil palm. In addition, local people have (illegally) converted their forest and agroforest areas to oil palm (Rist et al. 2010; Budidarsono et al. 2013).

**Table 1:** Basic characteristics of West Kotawaringin and Kapuas districts

	West Kotawaringin district	Kapuas district	Source
Area (km^2^)	8381	17,339	BPS, 2013
Population density (people/km^2^)	28	19	BPS, 2013
Annual population growth rate (%)	4.2	0.7	BPS, 2013
Per capita income (USD/year)	1860	1510	BPS, 2013
2010 forest cover (%)	52	74	MoF, 2010
Dominant forest use	Timber	Timber, NTFPs	Land-cover map 2010 (TBI Indonesia)
(Potential) land-use and land-cover change	Oil palm plantation (community and/or company scale)	Permanent agroforestry rubber, timber plantation	FGD in March 2014

Kapuas district is located in the south east of Central Kalimantan with a total area of 17,339 km^2^. Major land-use change in this district started from the establishment of a mega rice project in 1994/1995 that converted most of the peat forest to agriculture. This project was integrated with a transmigration programme that relocated many people from the islands of Java, Sumatra and Bali. In 2000/2001, the project was declared a failure leaving in its wake degraded peat forest and poverty. Many of the transmigrants have left the area resulting in a low annual population growth rate of 0.7 % and population density of about 19 people per km^2^ (Suyanto et al. 2009; Galudra et al. 2011; National Statistic Bureau (BPS) 2013). Forest is the main land cover with timber production and non-timber forest products (NTFP) as the main livelihoods.

### LUCES model

#### Model description

The LUCES model was designed to understand the decisions communities and private companies make in response to the forest moratorium policies and the subsequent effect on land-use and ecosystem service supply in the two study districts (West Kotawaringin and Kapuas). For the LUCES model, we adopted the Forest, Agroforest, Low-value Landscape Or Wasteland (FALLOW) model framework and the Land-Use Dynamic Simulator (LUDAS) model. The FALLOW model includes five main annual dynamic processes of biophysical and socioeconomic conditions of farmers and their decisions on land use (Mulia et al. 2013; Suyamto et al. 2009), while the LUDAS model includes spatio-temporal interactions in a human-landscape system (Le et al. 2008). The LUCES model was constructed for the simulation of 100 × 100 cells with input from land-cover maps provided by Tropenbos International Indonesia. The current version of the LUCES model was developed with a default plot size of 0.5 ha. This plot size is adjustable depending on the objective of the study and adjustments to input parameters. The LUCES model is coded in NetLogo 5.0.5, and the impacts of land-use strategies are presented as ecosystem services supplies. The ecosystem services in the LUCES model include six provisioning services (rattan (*Calamus* spp.), jelutong (*Dyera costulata*), timber (various species), rubber (*Hevea brasiliensis*), oil palm (*Elaeis guineensis*) and paddy (*Oryza sativa*)) and one regulating service (above and below ground C stocks). The decisions households make about land-use change are influenced by (1) the expectations of market prices based on past dynamics, (2) knowledge of the market and modes of production and (3) preferences for and perceptions of income and other benefits. The land-use decisions of private companies are mainly influenced by market prices and land zoning policies. The intended use of the LUCES model is for the ex ante evaluation of proposed land-use policies, e.g. the improvement and extension of the current forest moratorium. A detailed description and codes for the LUCES model can be obtained through the corresponding author or the World Agroforestry Centre (ICRAF).

#### Input maps and parameter values

The LUCES model requires inputs of spatial data and parameter values. The spatial data includes (1) land-cover maps, (2) maps of existing timber concessions and timber plantations, (3) maps of existing oil palm plantations and (4) maps of soil and plantation suitability. The parameter values used in the LUCES are related to economic, biophysical and demographic aspects. These include market prices, returns on land and labour, production, employment, demographics and ecosystem service supply. The maps and parameter values used in the LUCES model were obtained from different sources as explained in Table 2.

**Table 2:** List of data and parameters used in the LUCES model

Data	Year	Source
Land-cover map	1990, 2000, 2005, 2010	MoF, TBI Indonesia, ICRAF
Map of oil palm plantations (based on permit status)	2013	FNPF, OVI
Map of logging and forest plantation concessions	2010	MoF
Map of soil and plantation suitability	2012	Balittanah and ICRAF
Map of peat type and distribution	2010	Wetland International
Provincial spatial planning map	2003	Provincial government
Baseline map	2000	
Data on demography, production, prices, markets and employment at the subdistrict level	1990, 2000,2005, 2010	National Statistics Bureau
Ecosystem supply per land-use type	2010	Sumarga et al. 2014, 2015
Returns on land and labour	2010	Suwarno et al. 2016
Perceptions, learning, knowledge and selected agents for land change and ecosystem services	2012, 2013, 2014	Survey, personal communications, FGDs, scientific assumptions

#### Process overview and scheduling

The LUCES model is a spatially explicit representation of a land area (represented as a raster) with the potential for land-cover change in each pixel governed by a combination of formally planned and unplanned change. Private companies that obtained government permits drive planned land-use change, while households drive unplanned land-use change. Private companies change land use based on their interest in maximising profits, while households base their decisions on the income expectations of particular livelihood options (Abram et al. 2014). In the LUCES model, the livelihood options for local households include NTFP collection (rattan and jelutong) and the production of agroforestry rubber, paddy, oil palm and timber. Households will frequently change the current land use to agroforests, agriculture or oil palm plantation, while the decision on NTFP collection will not change the forest area.

**Fig. 2:**
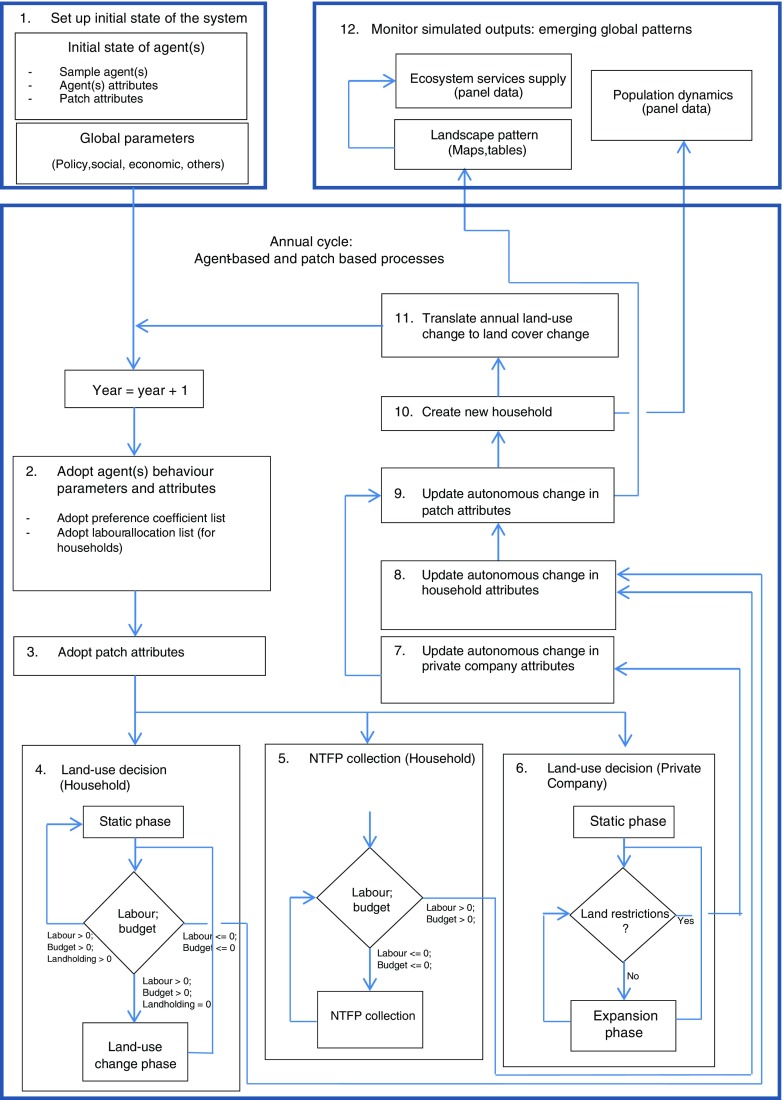
The main steps of the LUCES model simulation process for land-use decisions of households and private companies, as well as the impact of the land-use decisions on ecosystem service supply

The dynamic interactions in the LUCES model, under the simulation or scheduling programme, were developed based on a combination of the LUDAS model (Le et al. 2008; Le et al. 2010) and the FALLOW model (Suyamto et al. 2009). The scheduling programme consists of 12 steps, as presented in Fig. 2. The main time-loop of the simulation programme is an annual production cycle, which includes integrated patches of private company and household actions and decisions.

#### Scenarios and model simulations

In the forest moratorium scenario, we simulated the recent implementation of the forest conversion moratorium and two alternatives as follows (Table 3):

1. Business as usual (BAU) reflects the current trend, including the forest conversion moratorium, which initially ran from 2011 to 2014. The moratorium applies only to new or extended permits for companies converting peat forest to other land use; it does not apply to local communities.
2. The extended moratorium (EM) scenario extends the period of the forest conversion moratorium to 25 years starting from 2011. The forest conversion moratorium applies to new or extended permits for companies converting peat forest to other land use; it does not apply to local communities.
3. The moratorium plus livelihoods (MPL) scenario adds to the conventional moratorium an improved livelihood programme with enhanced markets for NTFPs, agroforestry products and community timber as well as an improved monitoring programme to avoid community logging.

**Table 3:** Key features of the three forest conversion moratorium scenarios using the LUCES model to determine current and future landscapes as well as ecosystem service supply

No.	Scenario	Description	Remarks
1	Business as usual (BAU)	- Protection for peat forest from conversion activities on a company scale (2011–2014) - Illegal conversion of peat forest on a community scale	- No change in road network and market prices is assumed during the 15 years simulation - Settlement distribution change based on the change in land demand and centre of economic activities
2	Extended moratorium (EM)	Similar to BAU, plus: - Extension of the period for protection of peat forest from conversion activities on a company scale (2011–2036) - New oil palm and timber plantations on a company scale can only be established on mineral soil	- Same as BAU
3	Moratorium plus livelihoods (MPL)	Similar to EM plus: - Increasing the market prices for NTFP, agroforestry products and community timber by about 15 % - Local demand for timber can only be supplied from community timber plantations	- Support the NTFP market chain, agroforestry products and community timber products - Increase illegal logging litigation - Other conditions are the same as BAU

#### Model validation

A validation test was used as an indication of the type of deviation that can be expected for the baseline predictions. Since LUCES is a complex human-environmental system model, its validity could not be achieved by a single test on point-to-point history matching. Hence, the model testing (Nguyen et al. 2007) was implemented to test (1) empirical verification and validation of the submodels and (2) rationality evaluation of the model structure. Further, we also applied backcasting and social validation approaches. The backcasting validation approach was applied to check similarities in patterns of simulated maps resulting from the model using reference maps (Pontius et al. 2008; Ray and Pijanowski 2010). Meanwhile, social validation was achieved through simulation results with key stakeholders in the two districts. In this simulation, we asked stakeholders to play the part of human agents (households and private companies) and the government as the legislator. Each group of agents (households and private companies) was allowed to make direct and indirect changes to land use based on their negotiations with other agents to meet their economic and conservation expectations. This simulation also included government regulations on forest and land-use management as the restrictive boundaries for agent groups in defining their land-use decisions.

## Results

### Land-cover output maps

**Fig. 3:**
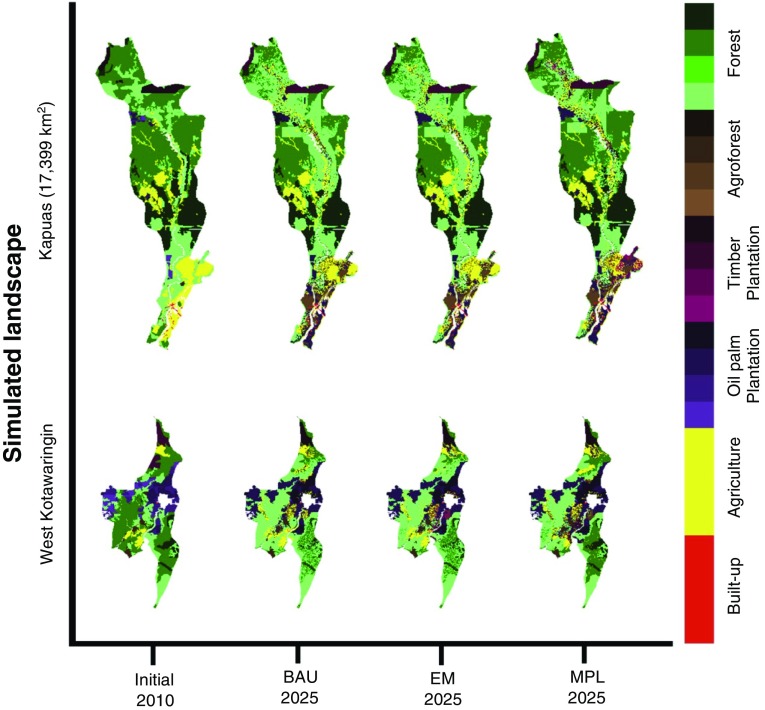
The dynamics of land-cover output resulting from the simulations of the LUCES model under three different scenarios

**Fig. 4:**
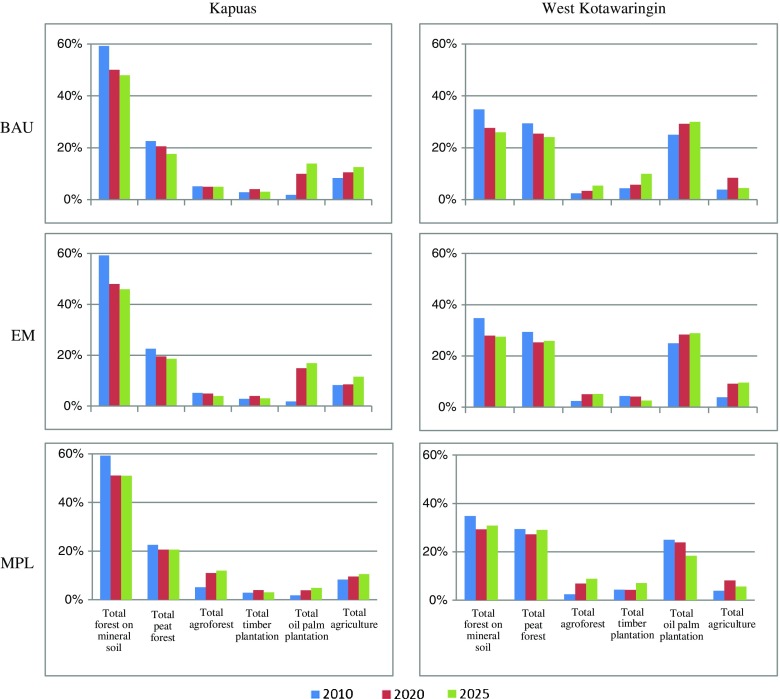
Simulated trends in land use as a percentage of the total area under three different scenarios. Similarity between simulated land use in 2010 (resulting from the LUCES model with the input of existing land use in 2005) and existing land use in 2010 are 64 % for Kapuas district and 62 % for West Kotawaringin district

Our simulations under the three different moratorium scenarios in West Kotawaringin and Kapuas districts show different patterns of land use in the last year of the simulation (2025) (Fig. 3). In West Kotawaringin, where the forests were under threat from the local communities and companies, the implementation of the BAU scenario from 2010 to 2025 could potentially reduce the area of peat forest and forest on mineral soil by about 11 and 5 %, respectively. Meanwhile, the implementation of this scenario could potentially increase the area of agroforests, timber plantation and oil palm plantation by about 2, 6 and 5 %, respectively (see Fig. 4). These increments are due to high unplanned land-use changes communities would have to make to meet their expected income. The EM scenario in this district does not provide any significant effort to reduce land-use change. The implementation of this scenario could also potentially decrease the area of peat forest and forest on mineral soil by about 7 and 3 %, respectively, and increase the area of agroforest, oil palm plantation and agriculture by about 2, 4 and 6 %, respectively. However, our simulation under the MPL scenario shows significant effort in decelerating land-use change. The area of forest on mineral soil decreased by about 4 % while the area of peat forest remained constant. This result shows that the implementation of this scenario could potentially decelerate conversion of forest on mineral soil and peat forest by about 6 and 5 %, respectively, compared with the BAU scenario (see Fig. 4).

Our simulations also show the reduction of forest on mineral soil and peat forest areas resulting from the implementation of the BAU scenario in Kapuas district by about 11 and 5 %, respectively, for the period 2010 to 2025. The implementation of the EM scenario in this district could significantly increase the loss of mineral soil forest and slightly decrease the loss of peat forest. Our simulation shows that the area of forest on mineral soil, peat forest and agroforest decreased by about 13, 4 and 1 %, respectively, while the area of oil palm plantation and agriculture increased by about 15 and 4 %, respectively. Contrary to the results for West Kotawaringin district, the implementation of the MPL scenario in Kapuas district only provides small differences in the dynamics of the forest on mineral soil and peat forest, which decreased by about 8 and 2 %, respectively. We found a significant increase in agroforest and a decrease in oil palm by about 7 and 9 %, respectively, compared with the BAU scenario. These land-use changes can be attributed to the availability of economic incentives for NTFP and agroforestry products that could increase the local income. Competitive incomes, comparable to the income from oil palm, have the potential to influence local community conservation of agroforest and forest areas and to slow down conversion to oil palm plantations.

### Ecosystem service supply

The results of the LUCES model show that, in general, the implementation of the MPL scenario provides better ecosystem service supply in Kapuas and West Kotawaringin. However, the results differ between the two districts due to the differences in land-use change patterns.

Our simulations for West Kotawaringin show that the implementation of the BAU scenario could potentially decrease the ecosystem service supply from forest (rattan, jelutong, timber and carbon sequestration) and agroforestry ecosystems (rubber). Extending the duration of the moratorium programme under EM scenario only provides insignificant improvements of the ecosystem service supply, and the rate of land-use change remains high. However, the implementation of the MPL scenario could potentially increase forest and agroforest areas and subsequently increase the supply of timber, rattan, jelutong, rubber and CO_2_ stock. The increase in the total CO_2_ stock (above and below ground, particularly in the peat) could significantly reduce potential CO_2_ emissions. The results of our simulation show that CO_2_ emissions could potentially be reduced by about 23 % through the implementation of the MPL scenario in this district. These results support the findings of Mulia et al. (2014) and Tata et al. (2015) that indicate the importance of economic incentives for NTFP collection in sustaining forest and agroforest areas, increasing the supply of rattan and jelutong and reducing potential CO_2_ emissions. Premium prices for NTFP, agroforestry rubber and community timber could change local perceptions of forest and agroforest conservation and subsequently reduce potential CO_2_ emissions from land-use change.

For Kapuas district, the results of our simulations show that the three scenarios for the forest moratorium policies are not significantly different with respect to the dynamics of ecosystem service supply for provisioning services. However, we found significant improvements in total CO_2_ stock under the MPL scenario that could consequently reduce potential CO_2_ emissions by about 15 %. This result indicates a strong correlation between the low population in this district and low expected income with low interest in land-use change and storing CO_2_. Another factor that influences this result is associated with the patterns of planned land-use change of private companies (see Table 4 for more information concerning this data on Kapuas and West Kotawaringin districts).

**Table 4:** The dynamics of ecosystem service supply under three different scenarios using the LUCES model

Ecosystem services (×1,000,000)										
Scenario	Timber (m3)	Rattan (ton)	Jelutong (ton)	Agroforest rubber (ton)	Rice (ton)	Oil palm (ton)	Above ground carbon (ton CO_2_e)	Peat carbon (ton CO_2_e)	Total carbon (ton CO_2_e)	Annual emissions (ton CO_2e_)
Kapuas district										
Initial 2010	43	0.8	0.4	0.01	0.5	0.08	759	2781	3540	
BAU 2025	40	0.8	0.3	0.3	0.4	1.7	721	2752	3446	6.3
EM 2025	41	0.8	0.4	0.4	0.3	1.6	730	2730	3460	6.1
MPL 2025	41	0.8	0.4	0.4	0.3	1.6	736	2726	3467	3.9
West Kotawaringin district										
Initial 2010	14	0.3	0.1	0.09	0.07	2	276	439	716	
BAU 2025	9	0.2	0.06	0.1	0.1	3.5	213	416	629	6.5
EM 2025	10	0.3	0.07	0.2	0.2	3.6	215	422	637	6.1
MPL 2025	15	0.6	0.09	0.4	0.1	2.7	234	457	691	5.1

The results in Table 4 are in line with the preferences of the stakeholders on land use obtained from a series of FGDs. The economic incentive options through premium prices for NTFPs and agroforestry products have changed local preferences. Local communities prefer to maintain the area as forest and agroforest instead of converting to oil palm. Meanwhile, private companies gave no response to this option, since it would have no significant impact on their profits. Private companies will follow the government regulations on land use and land management when expanding their area.

## Discussion

### Land-use scenarios, land-use change and ecosystem service supply

Land-use policies are a key determinant of stakeholders’ land-use decisions. Stakeholders respond differently to land-use policies in an effort to maximise the benefits they receive from certain land use (Brooks et al. 2014; Nelson et al. 2009; van Noordwijk et al. 2011). Their decision making is mainly influenced by their income expectations that are defined based on their knowledge and social networks (Berkes et al. 2000; Rogers 2004; Turnpenny et al. 2014). As shown in our simulations, forest moratorium policies in Indonesia influence stakeholders and their land-use decisions. However, we found that extending the period of the forest moratorium in its current form has little effect on land-use change in West Kotawaringin district due to the high-income expectations (from oil palm) of households and private companies. Extending the period of the moratorium only stops private companies from converting peat forests to oil palm but not households, since this regulation only applies to companies. High-income expectations for oil palm profitability have increased the households’ interest in expanding the oil palm area, including on peatland. Meanwhile, private companies tend to expand their oil palm plantations in degraded forest on mineral soil, since the regulations of the forest moratorium only apply to peat and natural forest. Moreover, it is often unclear if forests can be considered natural or degraded and government officials may not always have strong incentives to carry out a strict interpretation of the moratorium (Sloan 2014). Hence, the moratorium in the way it is currently implemented is not sufficiently effective to ensure a strong decline in forest loss (Margono et al. 2014) and to subsequently reduce potential CO_2_ emissions (Busch et al. 2015).

In Kapuas district, the lower population density and low expectations for oil palm performance have resulted in more stable land-use conditions. This result supports the empirical findings of Tachibana (2016) who highlighted population and expected income as the main drivers of land-use and land-cover change.

In our MPL scenario, we assumed that economic incentives for farmers/households were provided through premium prices for NTFPs, agroforestry rubber and community timber. We also assumed that the local government provides subsidies for producing these provisioning services through tax reductions. Based on these assumptions, our simulations show a greater increase in forest, agroforestry rubber and community timber plantation areas in West Kotawaringin and Kapuas districts, compared with the other two scenarios. The premium prices for NTFPs, agroforestry rubber and community timber have shifted the expectations of the households and changed their land-use decisions. Households tend to conserve more forest and agroforest areas that indirectly reduce potential CO_2_ from land-use change. This result supports other studies that found positive ecological effects when land-use scenarios that give priority to conservation and livelihoods were implemented (Mulia et al. 2013; Sunderland et al. 2008). However, premium prices did not change the expectations of the private companies concerning oil palm plantations, timber plantations and logging concessions.

### Policy implications

Terrestrial ecosystems, such as forests or managed agricultural lands, are subject to multiple natural processes and human interventions that have major effects on global climate (Carreño et al. 2012; Foley et al. 2005; Le et al. 2010). Reducing green house gas emissions and increasing CO_2_ sequestration in terrestrial ecosystems represents an important short-term option for mitigating global climate change. However, an array of policies to govern land-use changes is needed to achieve this. Considering the integration of climate change, sustainable development and poverty reduction, flagged in the Paris agreement, the implementation of such policies at the national level should articulate the integration of local livelihood programmes in a country’s strategic approaches (UNFCCC 2015a).

A wide scope of forest moratorium policies was part of the preparations for Indonesia’s INDCs to combat climate change and its impact on humans and ecosystems (Murniningtyas et al. 2015). Forest moratorium policies have recently been extended until 2016 and cover the suspension of permits for converting peat and secondary forests. However, the policy has yet to include a livelihood programme, as required in the Paris agreement. Considering local people as important stakeholders who may contribute to land-use change and global emissions, sustainable local livelihoods are important drivers of land use (Medrilzam et al. 2014; Sunderland et al. 2008; Tachibana 2016; van Noordwijk et al. 2008). As shown in our simulations, the option of including livelihood programmes in the MPL scenario could significantly decrease the rate of forest conversion in the two districts and indirectly reduce potential CO_2_ emissions. We also found that the model clearly depicts the multi-faceted nature of economic incentives in decelerating land-use change and restoring ecosystem benefits. The option of providing premium prices and cost subsidies for NTFP and permanent agroforestry production could increase potential local benefits. Equally, this scenario shows that premium prices (15 % higher than local prices) and cost subsidies (covering 5 % of production costs) have increased the benefits from NTFP and permanent agroforestry production to the level of benefits received from oil palm. This reduces local interest in converting forests and agroforests to oil palm and thus reduces local carbon emissions. These results support previous findings that the implementation of a conservation scenario will only work with a supporting programme that can promote ecosystem services as a viable livelihood option (Börner et al. 2011; McShane et al. 2011; Wunder 2013). The role of economic incentives in supporting the effectiveness of an environmental programme has also been shown by Kemkes et al. (2010), McCann (2013) and van Noordwijk et al. (2014).

The combination of conservation and livelihood programmes under the forest moratorium policy in West Kotawaringin and Kapuas districts could be achieved if traditional practices of tapping jelutong and agroforestry rubber were encouraged. These activities will potentially support local livelihoods that have had long experience and tradition in jelutong and agroforestry rubber tapping. From an ecological perspective, this option could potentially conserve and restore peat forest ecosystems and reduce emissions from LULUCF.

Considering Indonesia’s commitment to reduce emissions from LULUCF, the results of the LUCES model could provide an essential input for decision makers to develop additional programmes to improve the effectiveness of forest moratorium policies in decelerating land-use change and reducing CO_2_ emissions. The LUCES model, developed at the district level, could be scaled up to assess the implementation of forest moratorium policies nationally. Moreover, it could support national governments in evaluating and improving their strategies to mitigate global climate change as formulated in their INDCs.

## Conclusions

Our paper demonstrates how land-use decisions and ecosystem services can be modelled at the scale of Indonesian districts. We show that in West Kotawaringin district, the high economic value of oil palm has increased the communities’ interest in oil palm. Consequently, they are more likely to convert their forests and diverse agroforest areas to oil palm monocultures. However, the lower income expectations of communities in Kapuas district (achievable through NTFP and agroforest rubber production) have led to more conservation of forest and agroforest and hence a lower rate of land-use and land-cover change. Our simulations using the LUCES model show that it is important that the current forest moratorium is complemented with livelihood programmes that facilitate the generation of local income from forests that do not involve forest conversion. A moratorium with livelihood support could significantly reduce potential CO_2_ emissions from LULUCF by about 23 % in West Kotawaringin district and 15 % in Kapuas district. Hence, it is important to include sustainable livelihood programmes in the implementation of forest moratorium policies as a national mitigation strategy in the Indonesian INDC.

With the global relevance of these simulations in a high emission area such as our study sites, in Indonesia, we can see the need for forestry sector mitigation and adaptation strategies to include a specific focus on local livelihoods as agents that interact with government and large-scale plantations. The instruments a government uses to influence land-use decisions, and associated greenhouse gas emissions, will differ from large-scale operators to smallholders. The spatial planning route that adjusts, or temporarily stops as in a moratorium, permitted land-cover change can have an impact on the large-scale operators, provided that legality of their operations is checked and linked to their market access. To influence land-use choices of smallholders, however, removal of market barriers, taxes (such as the tax on forest products that also applies to harvests from private land in Indonesia) and depressing trade policies (as in those that affect farm-gate prices for rattan in Indonesia) may be essential. To achieve mitigation and adaptation, policies need to start with (holistic) local needs, rather than the stated (sectoral) policy objectives. A mindset is needed that accepts the de facto driving forces beyond what government plans normally include. Tools such as the one we present here can aid such processes.

## Figures and Tables

**Fig. 5:**
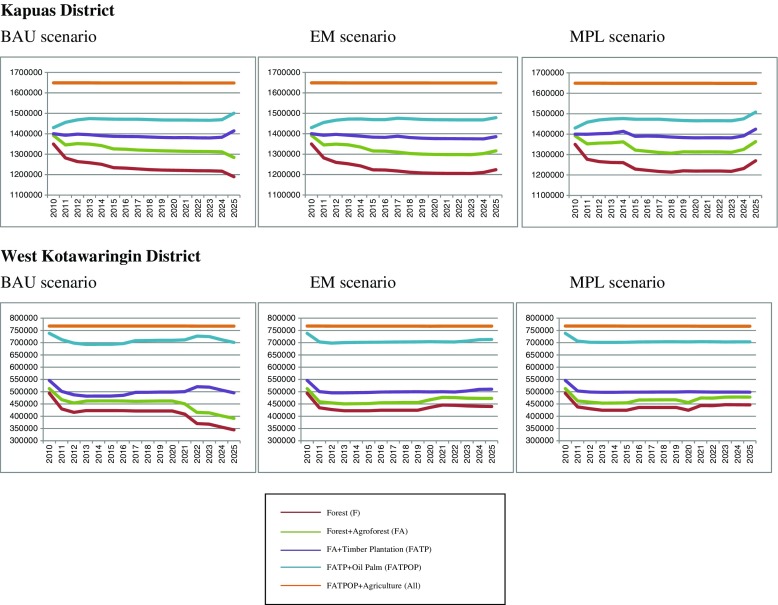
Trends in land-use change resulting from LUCES

## Appendix 1

**Table 5:** Household types and their α and β learning, degree of prioritisation, degree of sharing and radius of sharing networks used in the LUCES (adapted from van Noordwijk 2002; Rogers 2003; Suyamto et al. 2009; Mulia et al. 2013)

No.	Household types	Population fraction within the households	α learning (expectations adjusted rate of self-experience)	β learning (expectations adjusted rate of the experiences of others)	P (degree of prioritisation)	Degree of sharing network (other people or peers)	Radius of sharing network (km)
1.	Innovator	The one (1 %)	Very high (±1)		Very high (±2)	Very high (±10 people)	Very far (≥50 km)
2.	Early adopter	Minority (3 %)	High (±0.75)		High (±1.5)	High (±8 people)	Far (40–50 km)
3.	Early majority	Majority (45 %)	Medium (±0.5)		Proportional (±1)	Proportional (±6 people)	Medium (30–40 km)
4.	Late majority	Majority (45 %)	Low (±0.25)		Low (±0.5)	Low (±4 people)	Close (20–30 km)
5.	Laggard	Minority (≤ 6 %)	Very low (±0.1)		Very low (±0.25)	Very low (±2 people)	Very close (10–20 km)

α learning represents his/her own experiences, and β learning represents the experiences of others. Both α and β learning are assumed to contribute to a household’s economic expectations following the equations below:

*e*_*t*(own)_ = *e*_*t* − 1_ + *α*(*r*_*t* − 1_ − *e*_*t* − 1_) (1)

$$ {e}_{t\left(\mathrm{others}\right)}={e}_t+\beta \left({\overset{-}{e}}_t-{e}_t\right) $$ (2)

where *e*_*t*(own)_ and *e*_*t*(others)_ are adjusted expectations according to their own experiences and experiences of others, *e*_*t* − 1_ is the expectation of a given household (in € per person/day) at time *t* − 1, *r*_*t* − 1_ is the remuneration of a particular livelihood option currently earned by a given household (in € per person/day), and ${\overset{-}{e}}_t$ denotes the mean of the adjusted (at time *t*) expected wages of a particular livelihood option of other households (in € per person/day). The expectation adjustment rate is α (0 ≤ *α* ≤ 1), and β is the expectation adjustment rate of a given household’s experience of other households (0 ≤ *β* ≤ 1)

## Appendix 2

**Table 6:** Thirteen submodels of LUCES coded in NetLogo 5.0.5

Name	Brief functionalities/tasks	Involved entity
Initialisation	Import GIS data, population data and household data. Generate the first harvesting/planting plot of private company areas, create household-pixel links	Household pixels; private company pixels
Set labour requirements	Annually set the list of labour requirements for each household as community agents	Households
Choice of agricultural and agroforestry activities	Perform agricultural land-use (paddy field and oil palm plantation) choices; perform agroforestry land-use (rubber) choices. This step includes bounded-rational choices nested in rule-based decisions	Household pixels
Choice in NTFPs	Perform choice in NTFP collection. This step includes bounded-rational choices nested in rule based decisions on expected income	Household pixels
Update agent state	Annually update change in household and private company profiles	Households and private companies
Agent categorised	Annually categorise agents into the most similar groups	Households and private companies
Generate agent coefficients	Generate behaviour coefficients for agents, allow variants within groups and stabilise the behaviour structure of the group	Households and private companies
Forest yield dynamics	Calculate basal area for forest stands in response to human interventions (logging)	Pixels
Natural transition	Perform natural transition among vegetation types based on ecological edge effects	Pixels
Create new community households	Create new households controlled by empirical function and population	Households
Ecosystem services dynamics		
1. Provisioning service		
Paddy and oil palm production	Calculate the economic yield of paddy fields and oil palm plantations in response to human investment and site condition	Household and private company pixels
Agroforestry rubber production	Calculate the economic yield of agroforestry rubber in response to human investment and site	Household pixels
Rattan and Jelutong collection	Calculate potential yield of NTFPs based on the basal area of the forest stand	Household pixels
2. Regulating service		
Carbon sequestration	Calculate carbon stock and carbon emissions of each land-use type by assigning a time average for carbon density	Pixels

## Appendix 3

**Table 7:** Parameters of ecosystem service supply per land-cover type used in the LUCES model

Land-cover type	Succession	Time bound (years)	Stocks per hectare							
			Timber (m3/ha)	Rattan (ton/ha)	Jelutong (ton/ha)	Rubber (ton/ha)	Oil palm (ton/ha)	Paddy (ton/ha)	Above ground CO_2e_ (ton/ha)	Peat CO_2e_ (ton/ha)
Mineral-soil forest	Primary mineral-soil forest	100	60	1	0	0	0	0	926	0
	Old secondary mineral-soil forest	50	45	0.79	0	0	0	0	787	0
	Young secondary mineral-soil forest	25	20	0.25	0	0	0	0	411	0
	Pioneer mineral-soil forest	0	0	0.125	0	0	0	0	110	0
Peat forest	Primary peat forest	100	30	0	2	0	0	0	463	7000
	Old secondary peat forest	50	25	0	0.2	0	0	0	394	3500
	Young secondary peat forest	25	10	0	0.1	0	0	0	206	1250
	Pioneer peat forest	0	0	0	0.025	0	0	0	25	750
Agroforest	Post production agroforest	50	15	0	0	0.25	0	0	412	0
	Late production agroforest	25	12.5	0	0	4	0	0	410	0
	Early production agroforest	5	1	0	0	3	0	0	242	0
	Pioneer agroforest	0	0	0	0	0	0	0	25	0
Timber plantation	Mature timber plantation	10	30	0	0	0	0	0	515	0
	Pre harvest timber plantation	5	25	0	0	0	0	0	513	0
	Young timber plantation	2	5	0	0	0	0	0	303	0
	Pioneer timber plantation	0	0	0	0	0	0	0	31	0
Oil palm plantation	Post production oil palm plantation	25	0	0	0	0	17	0	206	0
	Late production oil palm plantation	10	0	0	0	0	24	0	205	0
	Early production oil palm plantation	5	0	0	0	0	10	0	112	0
	Pioneer oil palm plantation	0	0	0	0	0	0	0	12	0
Agriculture	Agriculture	0	0	0	0	0	0	3	0	0
